# Antioxidants in Poultry Nutrition and Reproduction: An Update

**DOI:** 10.3390/antiox9020105

**Published:** 2020-01-25

**Authors:** Peter F. Surai

**Affiliations:** 1 Vitagene and Health Research Centre, Bristol BS4 2RS, UK; psurai@feedfood.co.uk; 2 Department of Hygiene and Poultry Sciences, Moscow State Academy of Veterinary Medicine and Biotechnology named after K.I. Skryabin, Moscow 109472, Russia; 3 Department of Microbiology and Biochemistry, Faculty of Veterinary Medicine, Trakia University, Stara Zagora 6000, Bulgaria; 4 Department of Animal Nutrition, Faculty of Agricultural and Environmental Sciences, Szent Istvan University, H-2103 Gödöllo, Hungary; 5 Saint-Petersburg State Academy of Veterinary Medicine, St. Petersburg 196084, Russia

For the last three decades poultry production worldwide has made tremendous progress in terms of quantity and quality of meat and egg production, including improvement of growth rate and feed conversion rate. However, it has been proven that commercial poultry production is associated with a range of stresses including environmental, technological, nutritional, and internal/biological [1,2]. Indeed, it is practically impossible to avoid stresses, so therefore overproduction of free radicals and oxidative stress are very common problems in commercial poultry production. Natural antioxidants should, as a result, be included as an essential factor in poultry nutrition. 

This special issue consists of seven articles related to natural antioxidants in avian nutrition and reproduction. In particular, three papers were related to meat quality issues. In fact, meat quality includes four major categories: technological, nutritional, hygienic, and organoleptic parameters, and antioxidants affect three of them. Estevez and Petracci [3] evaluated the effect of Mg supplementation (0.3%) on production parameters, the redox status and meat quality in growing broilers. The author showed that Mg dietary supplementation increased Mg^2+^ concentration in blood and liver, while Mg^2+^ concentration in muscles was not affected. A very important finding of this paper is related to improvement of water-holding capacity (WHC) of the meat due to Mg dietary supplementation. Indeed, WHC plays a very important role not only in the improvement of meat appearance (major determinant of making decision for meat choice on the supermarket shelf), but also in meat juiciness and prevention of microbial spoilage. It was shown that dietary Mg supplementation was associated with improved AO defenses (catalase activity) in the liver, muscles, and blood and decreased protein oxidation marker (protein carbonyls) in the liver and plasma. 

One more important finding of this study is protective effects of Mg dietary supplementation on the development of two major myopathies affecting meat quality, namely white striping (WS) and wooden breast (WB), affecting 47% and 40% of birds in the study. Interestingly, meat from birds with WS and WB was characterized by decreased WHC with increased protein oxidation in WB-affected birds. There are several limitations of the study related to a comparatively small number of birds (32 chickens per treatment) and only one marker of protein oxidation (protein carbonyls) used. 

Previous publications of the authors made great contributions to the understanding detrimental effects of protein oxidation on meat quality and human health [4,5]. To address involvement of protein oxidation in the development of WB and WS it is necessary to conduct further research using more sensitive markers of protein oxidation with specific emphasis to cysteine and methionine groups in proteins which are associated with redox status of tissues. Furthermore, nutritional modulation of antioxidant system of the body to prevent WS and WB and improve meat quality awaits further investigation. Therefore, Shakeri et al. [6] studied the effects of betaine, or a mixture of betaine, selenized yeast, and vitamin E on growing birds under physiological conditions and cyclical heat stress (HS) conditions in a small university trial with 72 day-old birds grown for 42 days. The chickens were fed either a commercial control diet (50 IU/kg Vit. E and 0.3 mg/kg Se) (CON), or the control diet supplemented with 1 g/kg betaine (BET) or the control diet plus a combination of 1 g/kg BET with 0.3 mg/kg Se (as selenized yeast) and 200 IU/kg Vit. E. Final body weight was shown to be significantly decreased by HS, while BET showed protective effects. It was also found that HS was associated with reduced cooking loss and had no effect on drip loss. Again, BET was found to decrease the drip loss. Furthermore, HS reduced the myofibril fragmentation index and increased lipid peroxidation as evidenced by increased thiobarbituric acid reactive species (TBARS). Heat stress was shown to increase TBARS post slaughter (5.65 vs 3.60 , *p* < 0.001), whereas TBARS were decreased by BET (4.08 vs 5.72, *p* < 0.001). 

It should be mentioned that additional supplementation of organic Se and vitamin E did not show extra protective effects on major parameters studied, including lipid peroxidation index in stress condition, in comparison to BET supplemented birds. However, in thermoneutral conditions the antioxidant combination in the chicken diet showed additional protective effects (in comparison to BET alone) against lipid peroxidation in the breast muscle. Clearly, BET was shown to be protective under heat stress conditions in growing birds. Molecular mechanisms of antioxidant-related protective effects of BET under heat stress are still not very clear. However, the possibility of BET affecting activity of vitagenes [7], responsible for stress adaptation, deserves more attention. A failure of antioxidant combination to improve BET protective efficacy reflects restricted knowledge in this antioxidant area and indicates that there is a need for more research in the field of antioxidant interactions and redox regulation of the antioxidant-related genes/vitagenes. In the Jiang et al. [8] study, a total of 324 pairs of breeding pigeons were selected and allotted to nine treatments in a completely randomized design, and the birds were fed dietary treatments for 45 d, including a Met-deficient basal diet (BD, crude protein = 15%, Met = 0.25%) and BD + 0.15%, 0.30%, 0.45%, or 0.60% dl-Methionine (Met) or dl-methionyl-dl-methionine (dl-Met-Met) diets. As one can expect, compared to the Met-deficient diet fed to the BD group, dietary dl-Met or dl-Met-Met supplementation effectively increased most of studied parameters of meat production and quality. Interestingly, catalase activity, total superoxide dismutase (SOD) activity, and glutathione peroxidase (GPx) activity were increased, while malondialdehyde (MDA) concentration, drip loss and cooking loss of squabs were decreased. Moreover, dl-Met-Met was more effective than dl-Met in decreasing the drip loss and improving the antioxidant activity of the breast and thigh muscles of squabs. On the one hand, Met residues in proteins can act as endogenous antioxidants involved in redox balance maintenance and stress adaptation [9]. On the other hand, Met is an important precursor of GSH [10] and also involved in protein structure stabilization [11]. Clearly, there is a range of mechanisms by which Met deficiency can detrimentally affect the antioxidant defense network and an optimal Met dietary supplementation is a key for high chicken growth, development, and stress resistance. 

Among the many different stresses affecting growing chickens, mycotoxins are considered to be major unavoidable nutritional stressors. It seems likely that oxidative stress and changes in gene expression and cell redox signaling are major molecular mechanisms of the detrimental effects of mycotoxins on poultry [12]. Kovesi et al. [13] evaluated the effect of ochratoxin A (OTA; 106, 654 and 1126 µg/kg feed) exposure for 3 weeks on lipid peroxidation, GSH concentration and GPx activity, as well as expression of oxidative stress response-related (KEAP1, NRF2) and glutathione system (GPX3, GPX4, GSS, GSR) genes in chickens. It was shown that OTA imposed oxidative stress in the liver and kidney leading to lipid peroxidation (MDA). The main finding of the study is dysregulation of the antioxidant defense network in the case of OTA exposure. For example, as a result of the highest OTA dose exposure, GSH concentration was shown to be increased in blood plasma and in liver, but not in red blood cell hemolysates and the kidney. It is not clear if these changes in GSH are just a reflection of disbalance between synthesis and usage of GSH or there is a redistribution of this antioxidant between other tissues due to OTA intoxication. In fact, neither GSH content nor GPx activity increased systematically during the period of OTA exposure. Again, tissue-specific response to OTA was evident, since expression of the KEAP1 gene was up-regulated in the liver, but down-regulated in the kidney. At the same time, increased expression of NRF2 gene was evident in the liver and kidney at the highest OTA exposure. The complexity of the AO system dysregulation is confirmed by down-regulation of Nrf2 dependent genes, GPX3, GPX4, GSS, and GSR due to OTA exposure, showing differences at gene expression and protein synthesis levels of various antioxidants under OTA-induced stress conditions. It seems likely that dysregulation of the antioxidant defense network and disruption of redox balance and signaling are important steps in mycotoxin-related stresses. This is true not only for OTA (this paper), but also for T-2 toxin [14], aflatoxins [15] and other mycotoxin exposure singly or in combination [16]. Therefore, elucidation of molecular mechanisms of dysregulating actions of mycotoxins on the antioxidant defense network and search for an effective strategy to deal with mycotoxin-related nutritional stresses in poultry production warrants further research. An important addition to this Special Issue was the paper written by Moller et al. [17] which was devoted to evaluation of liver antioxidants in relation to beak morphology, gizzard size, and diet of the common eider *Somateria mollissima.* Data on antioxidant defense systems in wild birds are quite limited, but they can help to answer many questions related to the optimal dosage of dietary antioxidants in feed for domestic birds in commercial conditions of meat and egg production. The authors studied vitamin E, carotenoids, and coenzyme Q10 (CoQ10), which are major antioxidants in avian species [18]. It was found that the eider had a disproportionately large liver for its body size compared to other species, but the concentration of CoQ10 decreased with liver size. It was hypothesized that individuals with small livers for their body size are also those that have the smallest ability for synthesis of CoQ in response to oxidative stress. The authors showed that the concentration of antioxidants (total carotenoids, vitamin E and CoQ10) decreased among individuals with larger beaks suggesting that individuals with large beaks had difficulty acquiring sufficient concentrations of antioxidants from feed. Furthermore, eiders with high concentrations of total carotenoids and CoQ10 were shown to have small gizzards, suggesting that the processing of food by a large gizzard was associated with a low concentration of antioxidants [17]. Hu et al. [19] reviewed potential mechanisms underlying the protective effects of polyphenols on heat stressed poultry. 

The authors characterized protective effects of three polyphenolics; namely, resveratrol, curcumin (a yellow polyphenol extracted from the traditional Chinese medicine, zingiber plant), and epigallocatechin gallate (EGCG), the primary component of green tea extract. In general, for the last 30 years a great number of research papers has been published to show antioxidant properties of polyphenolic compounds. However, there are several important issues related to polyphenolic assimilation from plant materials in poultry, farm animals, and humans which strongly suggest that polyphenolics are not direct antioxidants in biological systems but rather indirectly affect antioxidant defense mechanisms [20]. First of all, absorption and assimilation of polyphenolic compounds from the diet is extremely low and therefore their concentration in target tissues is too low to show effective direct free radical scavenging activity. This means that antioxidant properties of many polyphenolic compounds shown in the model systems *in vitro* are related to concentrations which are not achievable in biological tissues. Furthermore, quick polyphenolic metabolism suggests that active compounds showing biological effects at the tissue/cell level might be different from those consumed with the diet. In addition, depending on conditions, polyphenolics can show antioxidant or mild pro-oxidant properties. Therefore, it was suggested that on the one hand polyphenolics could have substantial protective effect in the gut where their concentration could be quite high. On the other hand, polyphenolics could affect various transcription factors, including Nrf2 and NF-κB, and by doing so indirectly upregulate antioxidant defences. Therefore, Hu et al. [19] paid special attention to the polyphenolic protective action in the chicken gut. Indeed, improvement of antioxidant defense system, probably via activation of transcription factors, including Nrf2 and NF-κB, is a key mechanism of protective effect of polyphenolics in heat-stressed poultry.

Surai et al. [21] presented an update on the antioxidant defense system in poultry. In general, that update is summarized in Figure 1.

Mitochondria and phagocyte cells are major sources of reactive oxygen and nitrogen species (RONS). There are also various stress conditions in poultry production increasing RONS production. It is well appreciated that RONS play important roles as signaling molecules; however, when their concentration is above threshold level, they can cause damage to main biological molecules, including lipids, proteins, and DNA/RNA. As a result of RONS excess, a stress response program (Oxidative stress response, OSR) is activated. Since oxidative stress can cause a range of damages to various molecules, in addition to OSR, other stress response programs, including heat shock response (HSR), unfolded protein response (UPR), hypoxia-induced response (HIR), and DNA damage response, are also activated. This leads to activation of various transcription factors, including HSF1, Nrf2, NF-κB, FOXO, HIF, p53, and others. As a result of the upregulation of transcription factors, various genes, including vitagenes, are activated. In fact, activation of HSF increased production of HSP70, Nrf2 activation increased synthesis of SOD, HO-1, elements of thioredoxin and glutathione systems; and FOXO activation would cause increased expression of sirtuins. There is a complex system of interplay between vitagenes and transcription factors. In fact, some vitagenes, like SOD, are affected by several transcription factors including Nrf2, NF-κB, p53, etc. Since SOD is responsible for production of H2O2, major signaling RONS, control of its concentration is of paramount importance. Furthermore, products of vitagene activation, e.g., sirtuins, would affect expression and activity of some transcription factors, including Nrf2, NF-κB, FOXO, etc. Some vitagenes can be activated directly without transcription factor involvement. This includes transcriptional regulation of SOD in response to ROS as well as activation of sirtuins by changes in NAD+/NADH ratio. In general, redox homeostasis plays an important role in the regulation of antioxidant defences. RONS are also responsible for adaptive production/activation of other antioxidants, which are not included into vitagene family (e.g., CoQ, catalase, various selenoproteins, etc.) and they all responsible for redox balance maintenance, stress resistance and adaptation leading to good health, high immunocompetence, high productive and reproductive performance of poultry. However, when the antioxidant defense system, together with the vitagene network, are not able to prevent or repair damages imposed by RONS to biological molecules, other protective mechanisms including mitophagy, autophagy, apoptosis, necroptosis, and ferroptosis are dealing with terminally damaged molecules, organelles or cells. As a result of disrupted redox balance and accumulation of damages in cells/tissues, health-related problems, including low immunocompetence, appear. In addition, decreased productive and reproductive performance can cause heavy economic losses for poultry industry. Since it is almost impossible to avoid stresses in commercial poultry production, a search for nutritional means of antioxidant system modulation, including usage of increased doses of vitamin E [22], selenium [23], taurine [24] and polyphenolics [19,25], is on the agenda of many research groups world-wide. It seems likely that vitagene modulation by nutritional means (e.g., carnitine, betaine, silymarin, taurine, vitamin and minerals [7,21]) is a new strategy to prevent commercially relevant stresses and maintain high productive and reproductive performance of commercial poultry.

## Figures and Tables

**Figure 1:**
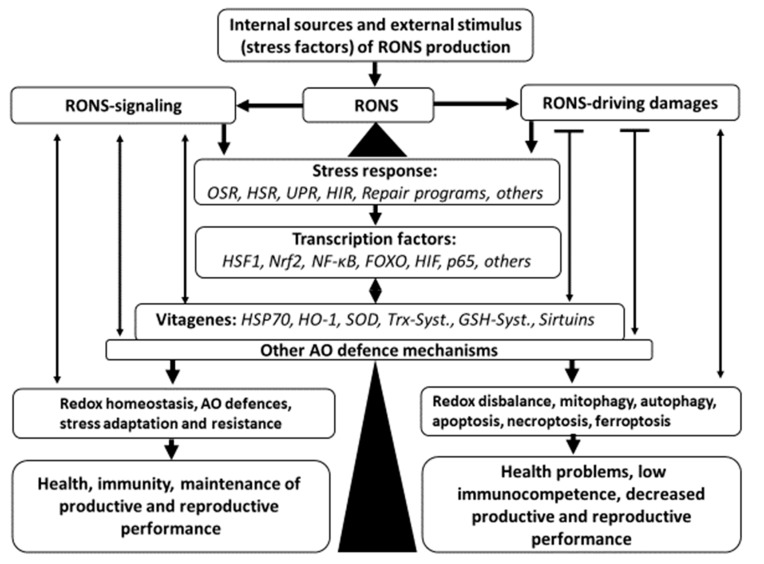
Antioxidant defense network in poultry (Adapted from [21]). (RONS- reactive oxygen and nitrogen species; OSR—oxidative stress response, HSR-heat shock response, UPR—unfolded protein response; HIR—hypoxia-induced response; HSF1—heat shock factor; Nrf2—transcription factor; NF-kB—transcription factor; FOXO—transcription factors; HIF—hypoxia-inducible transcription factor; p65—transcription factor; HSP70—heat shock protein 70; HO-1—heme oxygenase 1; SOD—superoxide dismutase; Trx-Syst.—thioredoxin system (thioredoxin/thioredoxin peroxidase (peroxiredoxins)/sulfiredoxin/thioredoxin reductase), GSH-syst.—glutathione system (glutathione/glutathione reductase/glutaredoxin/glutathione peroxidase).
